# Acute presentation of oncologic disease

**DOI:** 10.1186/1470-7330-14-S1-P25

**Published:** 2014-10-09

**Authors:** SE De Luca, E Casalini Vañek, C Carrera, JE Codas Thompson, MC Wirtz, EP Eyheremendy

**Affiliations:** 1 Hospital Alemán, Buenos Aires, Argentina

## 

The number of complicated oncologic patients is increasing accordingly to the high overall prevalence of cancer and the improved survival rate due to advances in cancer treatments. Progression disease and treatment-related complications are the most common causes. In this presentation, we review the pathophysiology of selected oncologic emergencies in which radiologists play a critical role in timely diagnosis and thus have a significant impact on patients’ care.

We selected patients who attended the emergency department of our hospital between 2010 to 2014 with different acute symptoms.

Of the oncological emergencies presented we found most commonly: compression of the spinal cord (4.5%), carcinomatous meningitis (13.6%); obstruction of the central airway (9%), oesophageal fistula (9%), pulmonary embolism (9%), superior vena cava syndrome (4.5%), pelvic abscess (18%), intra-abdominal haemorrhage (9.5%) and intestinal intussusception (13.6%).

Oncologic emergencies represent important causes of death in cancer patients. Radiologists should be familiar with imaging findings of acute conditions to optimize patient care. Recognition of key imaging findings allows prompt diagnosis and facilitates treatment, reducing morbidity and mortality with consequent better outcome.

